# Advances in Ni^I^/Ni^III^-Catalyzed C(sp^2^)–Heteroatom Cross-Couplings

**DOI:** 10.1021/acscatal.5c07964

**Published:** 2025-12-24

**Authors:** Aleksander R. Bena, Bartholomäus Pieber

**Affiliations:** Institute of Science and Technology Austria (ISTA), Am Campus 1, 3400 Klosterneuburg, Austria

**Keywords:** homogeneous catalysis, C(sp^2^)−heteroatom cross-coupling, nickel catalysis, oxidative addition, catalyst deactivation

## Abstract

C­(sp^2^)–heteroatom couplings operating via Ni^I^/Ni^III^ catalysis have emerged as an alternative to canonical Pd^0^/Pd^II^ systems that require complex ligand architectures. Despite intensive research efforts during the past decade, catalytic methods employing this approach are still mostly confined to activated starting materials and require high catalyst loadings due to the low catalytic activity of Ni^I^ and undesired catalyst deactivation events. This article highlights recent advances in the field toward solving these long-standing challenges. We survey strategies that streamline the generation of catalytically competent Ni^I^ species from bench-stable Ni^II^ precatalysts, and discuss mechanistic studies that shed light on deactivation pathways and the rate-determining oxidative addition of aryl halides. In the final section, we highlight recently developed synthetic methodologies, which provide evidence that limitations can indeed be addressed by working at elevated temperatures, employing alternative electrophiles, harnessing the benefits of additives, or fine-tuning the metal’s reactivity through the ligand field.

## Graphical abstract

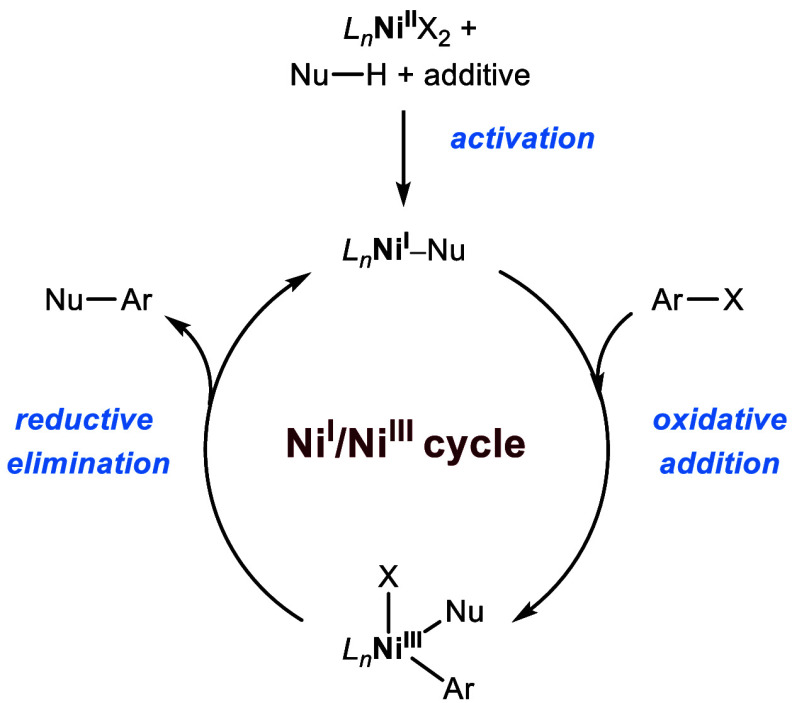

## 1 Introduction

Transition-metal-catalyzed C­(sp^2^)–C­(sp^2^) and C­(sp^2^)–heteroatom cross-couplings through the canonical M^0^/M^II^ cycle are a cornerstone of modern organic synthesis.^1^ The use of palladium catalysts has dominated the field since the 1970s and culminated in the Nobel Prize in Chemistry for Akira Suzuki, Ei-ichi Negishi, and Richard F. Heck in 2010.^2,3^ A key factor underlying the success of Pd-catalyzed cross-coupling reactions is the well-established catalytic manifold comprising oxidative addition (OA), transmetalation (TM), and reductive elimination (RE). This mechanistic framework has served as a foundational paradigm, enabling chemists to rationalize and predict reactivity, selectivity, and other critical aspects of cross-coupling processes. A crucial aspect in these reactions is the rational design of ancillary ligands, which enable couplings of challenging substrates, improve selectivity, and trigger cross-coupling under mild conditions and with low catalyst loadings.^4^ However, the steric and electronic ligand demands for OA and RE are often orthogonal, imposing intrinsic limitations (Figure 1A).^5^

**Figure 1:**
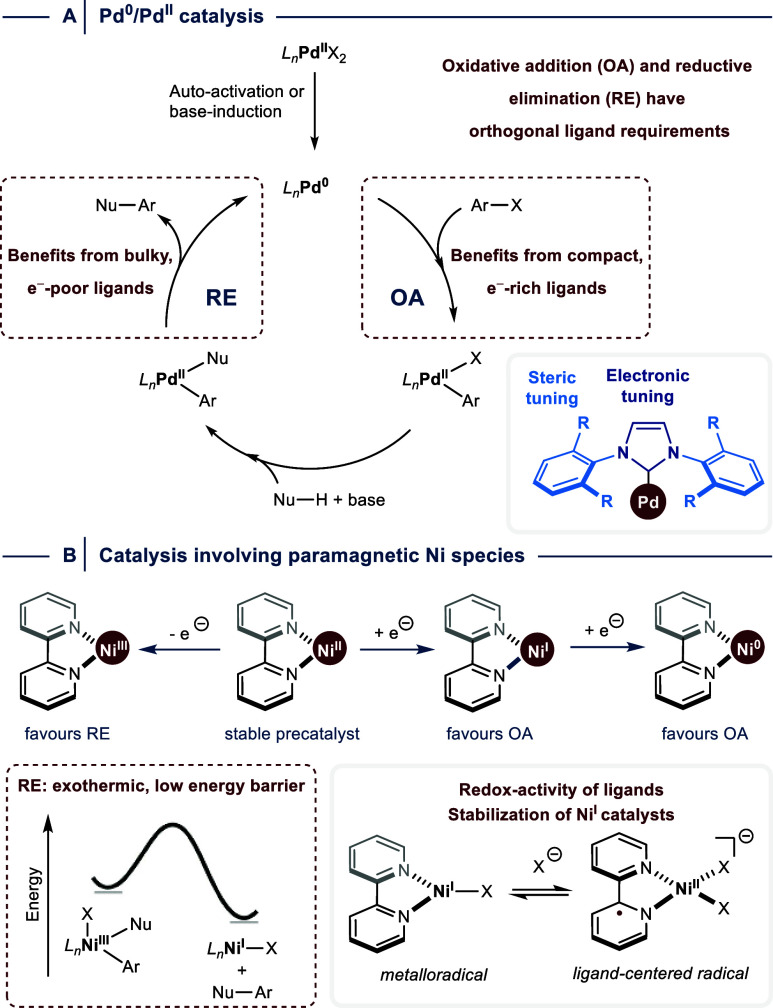
Facilitating elementary steps in cross-coupling catalysis. (A) Modulation of the metals’ reactivity in Pd^0^/Pd^II^ catalysis through the ligand field. (B) Oxidation state manipulation in catalysis involving Ni^I^ and Ni^III^ species. OA = oxidative addition. RE = reductive elimination.

The scarcity and cost of palladium make the development of catalytic systems based on earth-abundant base metals a desirable goal, especially for industrial applications.^1^ Moreover, in pharmaceutical applications, the amount of palladium permitted in drugs must be tightly controlled at low levels.^6^ In this context, nickel has emerged as an attractive alternative to palladium, offering not only economic and sustainability advantages but also distinct and versatile reactivity arising from the accessibility of multiple oxidation states (Ni^0^, Ni^I^, Ni^II^, Ni^III^) and its ability to engage in both two-electron and single-electron pathways.^7,8^ These features enable transformations that are often challenging or inaccessible with palladium, including couplings of alkyl substrates^9^ and cross-electrophile couplings.^10^

The combination of nickel catalysis with photocatalysis or electrochemistry has become an increasingly popular strategy for harnessing nickel’s potential in cross-coupling catalysis.^11–13^ Instead of modulating the metal’s ligand field, these dual catalytic transformations are orchestrated by manipulating the oxidation state of nickel through single electron transfer (SET) events that access paramagnetic Ni^I^ and Ni^III^ species that can undergo OA and RE, respectively (Figure 1B). Stabilization of these metalloradicals requires *N*-ligands, typically 2,2′-bipyridines, that confer redox activity by accepting an electron into the π* orbital.^14^

In contrast to Pd^0^/Pd^II^ catalysis, Ni^I^/Ni^III^-catalyzed carbon–carbon and carbon–heteroatom cross-couplings do not operate through the same sequences of elementary steps. The formation of C­(sp^2^)–C­(sp^3^) bonds between aryl halides and alkyl trifluoroborates or carboxylic acids was proposed to begin with the reduction of a Ni­(dtbbpy)­X_2_ (X = Cl, Br) precatalyst through two single electron transfer events, resulting in a Ni^0^ species (Figure 2A).^15^ The low-valent Ni catalyst is capable of trapping an alkyl radical generated through an off-cycle single-electron oxidation of the nucleophile (termed single-electron transmetalation), yielding a (dtbbpy)­Ni^I^ alkyl intermediate. This species undergoes efficient OA with a wide range of electrophiles, including aryl chlorides and pseudohalides, followed by RE of the desired product.^16,17^ Alternatively, Ni^0^ may first engage in OA followed by reaction with an organic open-shell species.^18^ Further, Ni^I^ halides were proposed to undergo transmetalation with trifluoroborates in the absence of a species that can induce radical generation through SET oxidation.^19^

**Figure 2:**
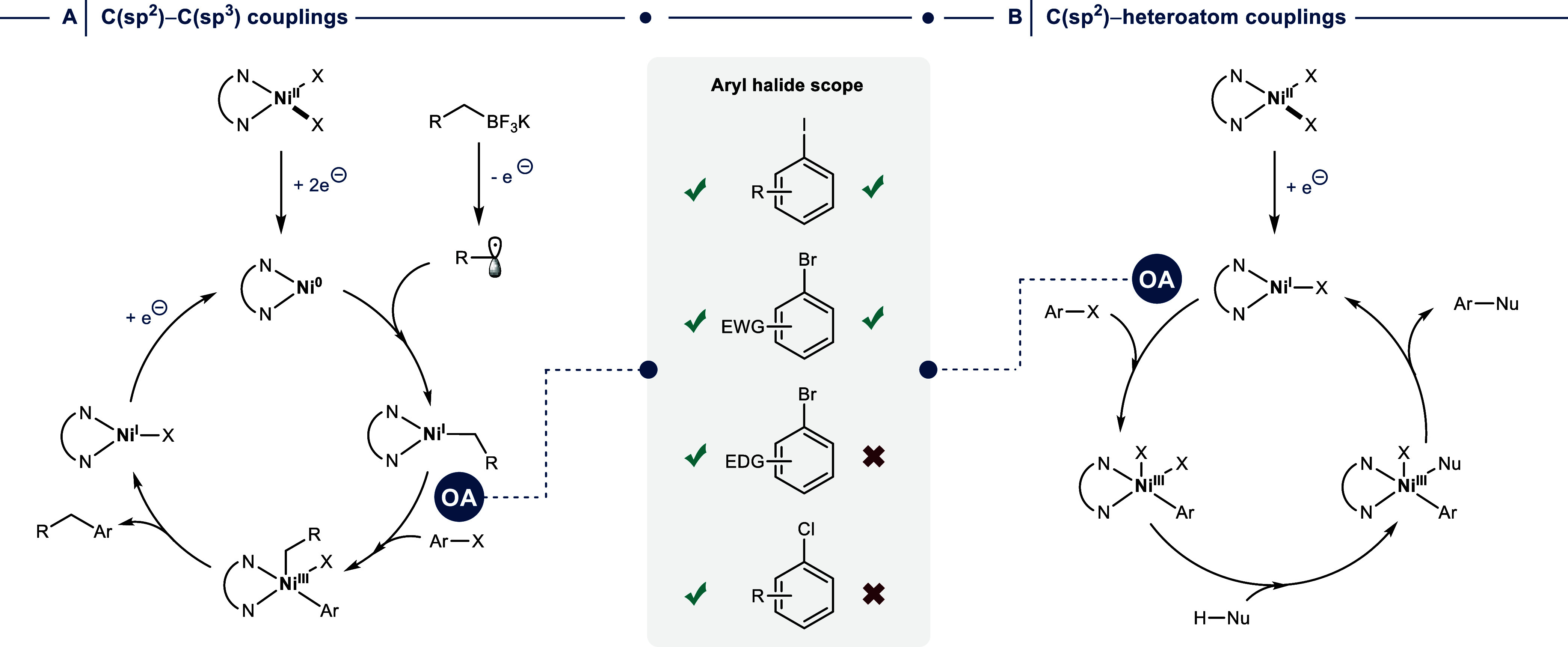
Proposed mechanisms of (A) C­(sp^2^)–C­(sp^3^) and (B) C­(sp^2^)–heteroatom cross-couplings via catalysis involving Ni^I^ and Ni^III^ intermediates explain the limited electrophile scope in the case of C­(sp^2^)–heteroatom cross-couplings. OA = oxidative addition. EWG = electron withdrawing group. EDG = electron donating group.

In the case of nickel-catalyzed C­(sp^2^)–heteroatom cross-couplings, the mechanism was initially also proposed to begin with OA of a photocatalytically generated Ni^0^ species to an aryl halide.^20,21^ However, mechanistic investigations by several research groups have provided convincing evidence that these reactions proceed through a Ni^I^/Ni^III^ catalytic cycle (Figure 2B).^22–26^ This mechanistic framework accounts for the limited electrophile scope, which is often confined to activated aryl halides such as aryl iodides and electron-deficient aryl bromides.^27^ In contrast to the catalytically potent Ni^I^ alkyl species operative in C­(sp^2^)–C­(sp^3^) couplings, the corresponding Ni^I^ halide intermediates in C­(sp^2^)–heteroatom couplings display lower nucleophilicity, which is detrimental for OA. This reactivity problem, in combination with the undesired formation of resting states,^23^ and catalyst deactivation through the generation of inactive dimers,^25,28^ and Ni-black,^26,29^ is arguably the reason for the high nickel catalyst loadings required in most protocols.

Despite the above-discussed limitations associated with the Ni^I^/Ni^III^ manifold, this mechanistic regime offers a distinct advantage over canonical Pd^0^/Pd^II^-catalyzed C­(sp^2^)–heteroatom cross-couplings. The bond-forming RE from high-valent Ni^III^ intermediates is generally exothermic and proceeds with a low activation barrier. In contrast, RE from Pd is often endergonic, or suffers from high transition state energies, requiring specifically designed ligand frameworks to facilitate this bond-forming step.^27^ Furthermore, Ni^I^/Ni^III^ catalysis enables efficient coupling of diverse *N*, *P*, *S*, and *O*-nucleophiles, thereby broadening synthetic utility. As a consequence, catalysis involving paramagnetic nickel species has the potential to streamline and generalize catalytic C­(sp^2^)–heteroatom bond formations.

In this Perspective, we provide an overview of recent advances in Ni^I^/Ni^III^-catalyzed C­(sp^2^)–heteroatom cross-couplings. First, we survey alternatives to common approaches that combine nickel catalysis with exogenous photoredox catalysts or electrochemistry. Further, we discuss mechanistic studies that shed light on deactivation pathways and the impact of ligand modifications. In the last section, we highlight strategies that aim to expand the substrate scope, which is crucial to maturing this approach into a robust catalysis platform able to compete—and potentially surpass—state-of-the-art M^0^/M^II^ methodologies.

## 2 Precatalyst Activation

### 2.1 Heterogeneous Reductants

The vast majority of methods that apply Ni^I^/Ni^III^ catalysis for C­(sp^2^)–heteroatom cross-couplings apply photoredox catalysis^12^ or electrochemistry^11,30^ to access the key Ni^I^ species from Ni^II^ precatalysts (Figure 3A). This requires complex catalytic cocktails or electrochemical setups and may cause catalyst deactivation due to undesired over-reduction to Ni^0^, especially in the absence of stabilizing ligands.^26,29^

**Figure 3:**
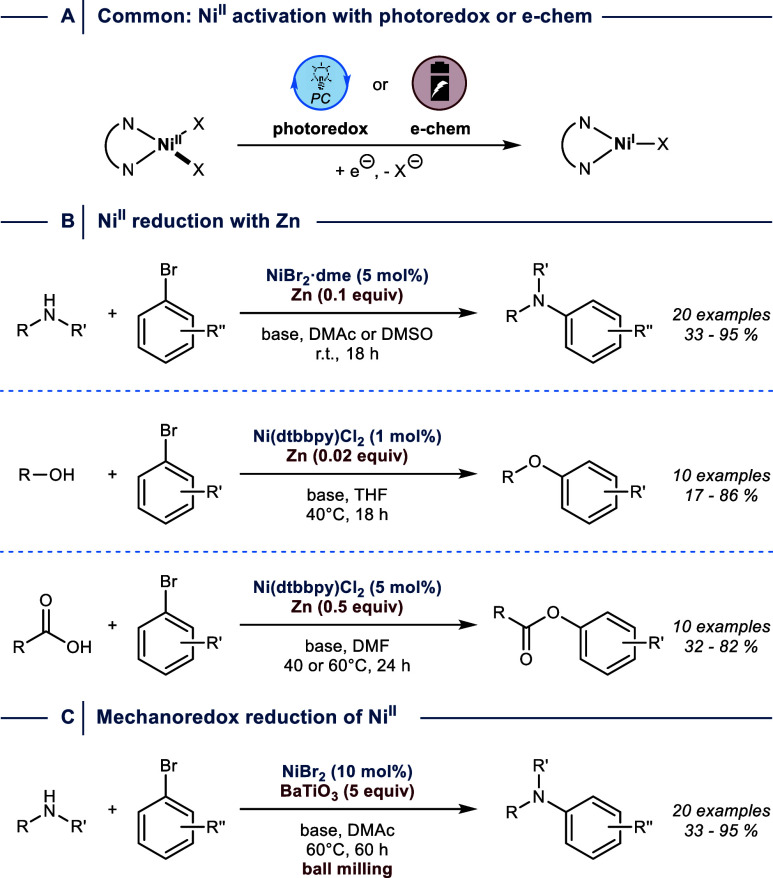
Initiation of Ni^I^/Ni^III^ catalysis. (A) Common approaches apply exogenous photoredox catalysis or electrochemistry. Precatalyst activation can be achieved using (B) substoichiometric amounts of Zn or (C) mechanical activation of piezoelectric materials. r.t. = room temperature. dme = 1,2-dimethoxyethane. dtbbpy = 4,4′-di-*tert*-butyl-2,2′-dipyridyl.

Alternatively, Nocera’s group demonstrated that Ni^I^/Ni^III^-catalyzed C­(sp^2^)–heteroatom cross-couplings of amines, alcohols, and carboxylic acids can be triggered using a Ni^II^ precatalyst in combination with substoichiometric amounts of zinc (Figure 3B),^31^ which, depending on the conditions, is not sufficiently reducing to generate Ni^0^.^32^ Indeed, when a Ni^0^ source was used instead of Ni^II^ in a zinc-free control experiment, low yields of the corresponding cross-coupling products were obtained. This observation suggests that Ni^II^ aryl halide species formed upon OA between Ni^0^ and the electrophile are not efficiently undergoing RE. Efficient catalysis can be observed using Ni^0^ and Zn, because the metal reduces Ni^II^ aryl halides formed upon OA to access the Ni^I^/Ni^III^ manifold.

More recently, BaTiO_3_ has been employed to initiate Ni^I^/Ni^III^-catalyzed C­(sp^2^)–N bond cross-coupling under mechanochemical conditions (Figure 3C).^33^ Upon mechanical force, the piezoelectric material becomes temporarily polarized enabling single electron reduction (mechanoredox) of donor molecules such as Ni^II^ precatalysts. The advantage of this approach compared to methods that initiate Ni^I^/Ni^III^ cycles via photoredox catalysis was demonstrated for the coupling of a polyaromatic compound that can engage in triplet–triplet energy transfer with the excited iridium photocatalyst, suppressing the cross-coupling reaction. Mechanoredox catalysis, on the contrary, resulted in the desired product in excellent yield.

### 2.2 Generation of Ni^I^ from a Sacrificial Ni Anode

As discussed above, Ni^I^/Ni^III^ catalyzed cross-couplings typically apply well-defined precatalysts that undergo single electron transfer reduction to initiate catalysis. To simplify electrochemically mediated C­(sp^2^)–N bond formations by avoiding the use of hygroscopic Ni^II^ precatalysts, Léonel and co-workers showed that a sacrificial Ni anode can serve as an alternative precatalyst (Figure 4A).^34^ This approach efficiently couples a range of cyclic secondary amines with aryl bromides using constant current conditions in an undivided cell setup (Figure 4B). Notably, a control experiment using a divided cell gave no cross-coupling product, which provides evidence that a cooperative process between both electrodes is necessary to generate the catalytically competent Ni species.

**Figure 4:**
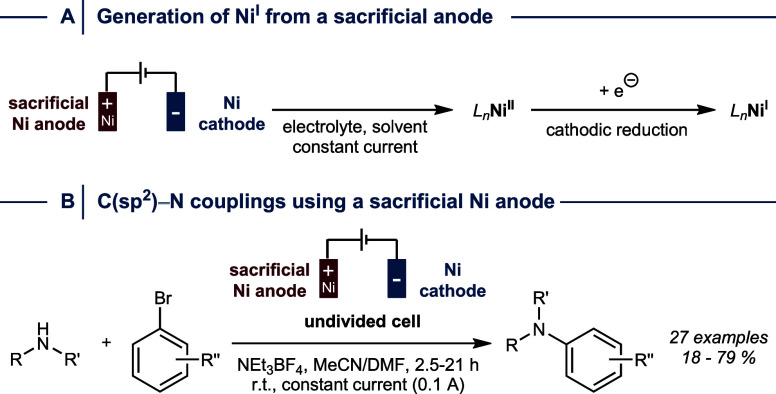
Generation of Ni^I^ from a sacrificial Ni anode (A) and its application for electrochemically mediated C­(sp^2^)–N cross-coupling catalysis (B). r.t. = room temperature.

In a follow-up study the same group demonstrated that the release of overstoichiometric Ni from the sacrificial anode can be avoided by first generating catalytic amounts of Ni from a sacrificial anode followed by exchanging the electrode to a platinum grid for conducting the cross-coupling reaction.^35^ Interestingly, this also enabled expanding the scope to primary and acyclic secondary amines.

### 2.3 Bifunctional Catalysts

Light-mediated Ni^I^/Ni^III^ catalysis can be triggered by incorporating the photoredox catalyst directly into the ligand architecture.^36^ This strategy ensures close spatial proximity between the excited photocatalyst and the Ni^II^ precatalyst, which addresses problems associated with the diffusion-limited bimolecular interaction between these two species.

Pignataro and co-workers realized this by covalently linking 1,2,3,5-tetrakis­(carbazol-9-yl)-4,6-dicyanobenzene (4CzIPN), a well-established organic photoredox catalyst, with a 2,2′-bipyridine ligand (Figure 5A).^37^ This design significantly improved catalytic efficiency for the coupling of various primary and secondary alcohols with electron-poor to moderately electron-rich aryl bromides. Remarkably, this approach enabled cross-couplings at low catalyst loadings (0.5 mol %) in the case of activated electrophiles.

**Figure 5:**
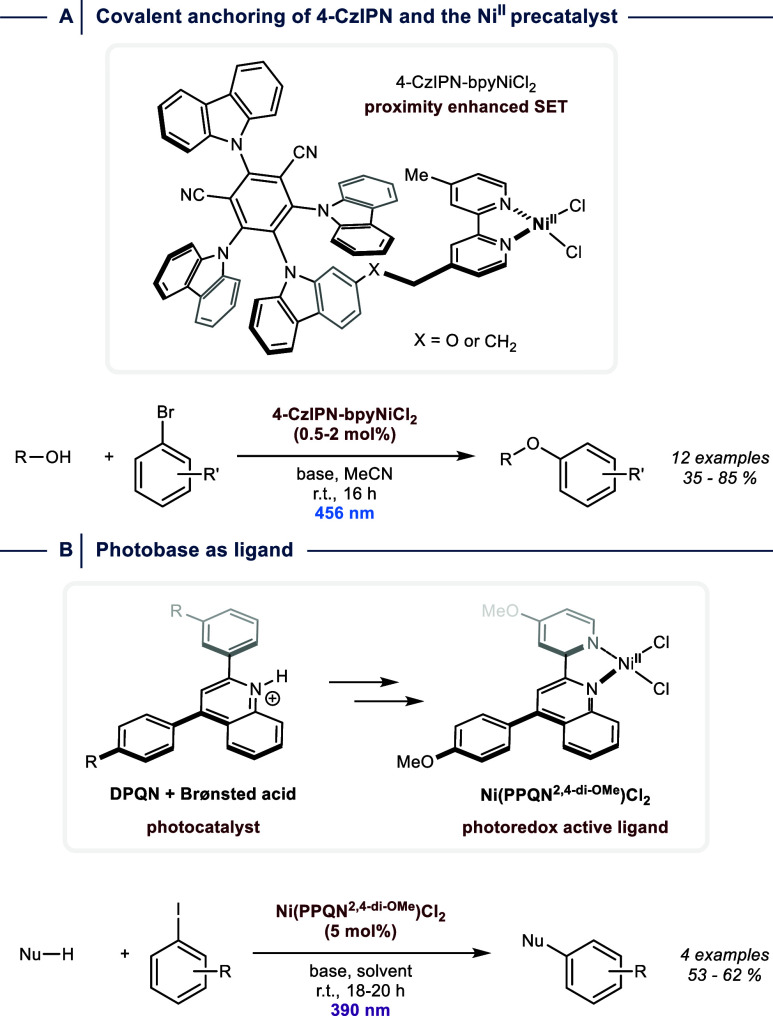
Incorporation of photoredox catalysts into the ligand architecture. (A) Attaching a photoredox catalyst to the bipyridine motif (B) Ligand design based on a quinoline photobase. SET = single electron transfer. r.t. = room temperature.

Li and co-workers followed a different strategy by integrating a quinolinium photoredox catalyst into the bipyridine ligand scaffold (Figure 5B).^38^ In combination with NiCl_2_ and a 390 nm light source, this photoredox active ligand facilitates several transformations, including C­(sp^2^)–N, C­(sp^2^)–O, C­(sp^2^)–S, and C­(sp^2^)–P couplings using aryl iodides. Similarly, cross-coupling catalysis can be carried out by integrating Ni^II^-bipyridine motifs into photocatalytically active polymers, such as covalent organic frameworks, or metal organic frameworks that contain a heterogenized photoredox catalyst.^39–41^

### 2.4 Direct Activation of Ni^II^ Complexes

In 2018, Miyake and co-workers uncovered that C­(sp^2^)–N couplings between activated aryl bromides and cyclic secondary amines using NiBr_2_ as a precatalyst proceed in the absence of an exogenous photocatalyst, metal reductant, or electrochemical conditions when 365 nm LEDs are used.^42^ Mechanistic studies suggested that excitation of an (amine)*_n_* NiBr_2_ species triggers a photoinduced electron transfer from the coordinated amines to the electron-poor Ni^II^ metal center, resulting in Ni^I^ formation. Similarly, it was shown that light-mediated C­(sp^2^)–O couplings can be carried out with catalytic amounts of NiBr_2_ in the presence of Cy_2_NMe.^43^

More recently, several well-defined photoactive Ni^II^ bipyridine complexes were developed as an emerging alternative to approaches that leverage single-electron transfer events between precatalysts and reductants (Figure 6A). For example, Doyle and colleagues have demonstrated that Ni^II^(dtbbpy) aryl halide complexes produce Ni^I^ upon irradiation with visible light.^44,45^ Direct excitation generates a metal-to-ligand charge transfer (MLCT) state that transitions to a triplet metal-centered d-d state,^44^ or a ligand-to-metal charge transfer (LMCT) state,^46,47^ resulting in homolysis of the Ni^II^–aryl bond. These complexes have been applied as precatalysts for C­(sp^2^)–O and C­(sp^2^)–N cross-couplings using 390 nm irradiation.^48,49^

**Figure 6:**
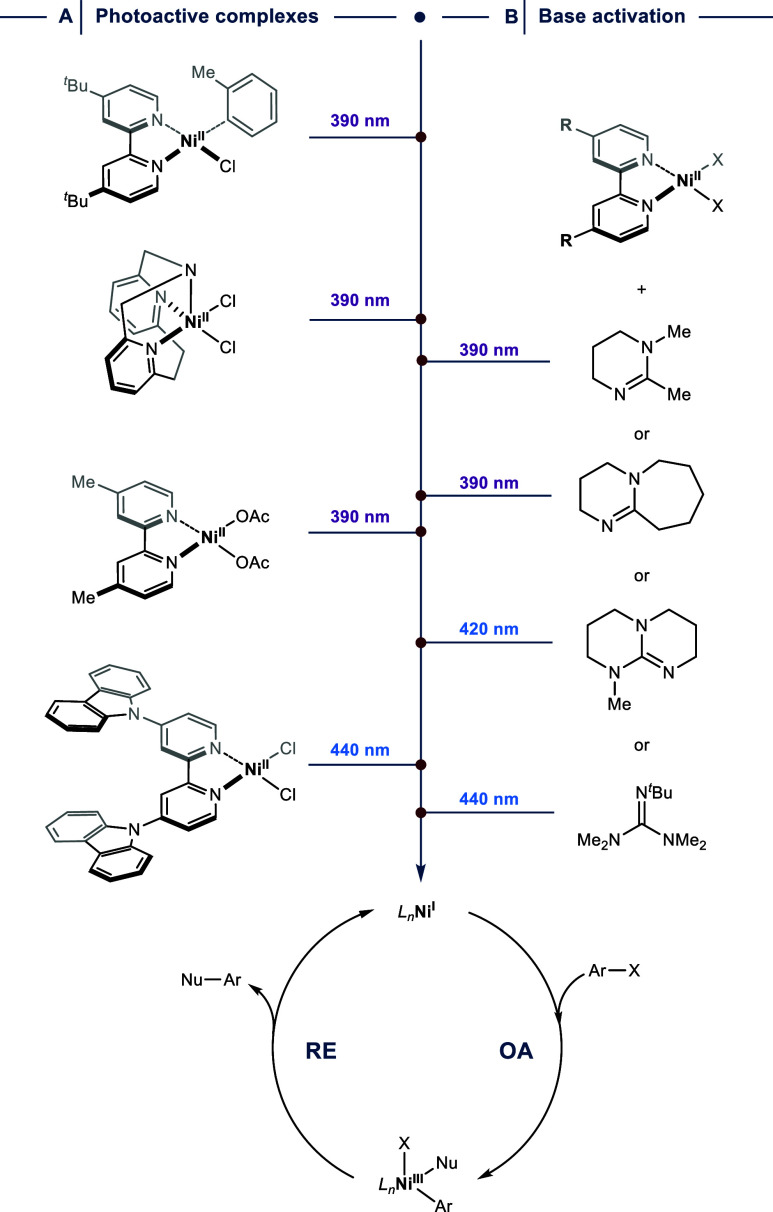
Photocatalyst-free generation of Ni^I^ from Ni^II^ precatalysts. (A) Photoactive Ni^II^ complexes. (B) The combination of Ni­(^R^bpy)­X_2_ complexes with amidine and guanidine bases results in the formation of photochemically active species. OA = oxidative addition. RE = reductive elimination.

The Mirica group has demonstrated that Ni^II^–Cl bond fission can occur in the case of a Ni^II^ complex bearing a tridentate pyridinophane ligand upon irradiation with purple LEDs.^50^ Mechanistic investigations indicated an initial population of the MLCT/LLCT state followed by relaxation to a metal-centered d-d state, which promotes cleavage of the metal–halogen bond. Remarkably, 0.2 mol % of Ni­(pyridinophane)­Cl_2_ were sufficient to catalyze the C­(sp^2^)–O coupling between MeOH and an activated aryl halide within 24 h under photocatalyst-free conditions. The photoactive nickel complex also enabled probing the key elementary steps of the Ni^I^/Ni^III^ catalytic cycle.

Xue and colleagues showed that (dMebpy)­Ni­(OAc)_2_ yields a catalytically competent Ni^I^ species for C­(sp^2^)–N cross-couplings upon irradiation with purple LEDs.^51,52^ Electron paramagnetic resonance (EPR) spectroscopy experiments in the presence of *N-tert*-butyl-α-phenylnitrone (PBN) provided strong evidence for the formation of an OAc radical, indicating that absorption of photons triggers homolytic Ni–O bond fission.

The Pieber lab developed Ni^II^ complexes equipped with donor–acceptor (D-A) ligands that harness low-energy visible light (440 nm) through an intraligand charge transfer (ILCT) transition by installing carbazole groups on 2,2′-bipyridines.^19,53^ Femtosecond-resolved optical transient absorption (OTA) experiments and time-dependent DFT studies suggested that the Ni­(Czbpy)­X_2_ (X = Br, Cl) complexes undergo fast intersystem crossing from a singlet to a triplet ILCT state that decays into an optically dark excited-state manifold followed by Ni^I^ formation. The position of the carbazole groups was shown to impact OA reactivity of the resulting Ni^I^ species.^19^ While the C­(sp^2^)–S coupling between a sodium sulfinate nucleophile and an electron-poor aryl bromide resulted in only small amounts of the desired product using 5 mol % of Ni­(5,5′-Czbpy)­Cl_2_, good isolated yields were obtained using only 1 mol % of Ni­(4,4′-Czbpy)­Cl_2_.

Notably, several recent reports showed that a combination of Ni­(^R^bpy)­X_2_ (X = Br, Cl) complexes and amidine or guanidine bases results in the formation of elusive species that engage with photons emitted from purple or blue LEDs, yielding Ni^I^ (Figure 6B).^54–57^ Although the underlying mechanism remains unclear, this approach potentially provides a highly general means of converting bench-stable (^R^bpy)­Ni^II^ halide complexes into catalytically competent Ni^I^ species. Similarly, thiols^58^ and diaryl phosphine oxides^59^ were proposed to coordinate to Ni­(dtbbpy)­X_2_ (X = I, Br) precatalysts, enabling Ni^I^ generation by direct excitation.

## 3 Mechanistic Insights

### 3.1 Catalyst Deactivation

Ni^I^/Ni^III^-catalyzed cross-couplings typically require high precatalyst loadings (up to 10 mol %),^60^ whereas the Pd^0^/Pd^II^ manifold can operate effectively with molar catalyst concentrations in the ppm to ppb range.^61,62^ Similar to endowing nickel with unique possibilities for catalysis, its ability to readily access paramagnetic oxidation states and engage not only in two- but also one-electron processes is responsible for unproductive pathways that compete with the desired catalytic cycle, resulting in low turnover numbers.

For example, oxidative deactivation of Ni^I^ through SET events results in the formation of Ni^II^ resting states that are similar or identical to the respective precatalysts (Figure 7A).^23,24^ As a result, Ni^I^/Ni^III^ catalytic C­(sp^2^)–heteroatom cross-couplings typically do not perpetuate in the absence of reducing species that reinitiate the catalytic cycle.

**Figure 7:**
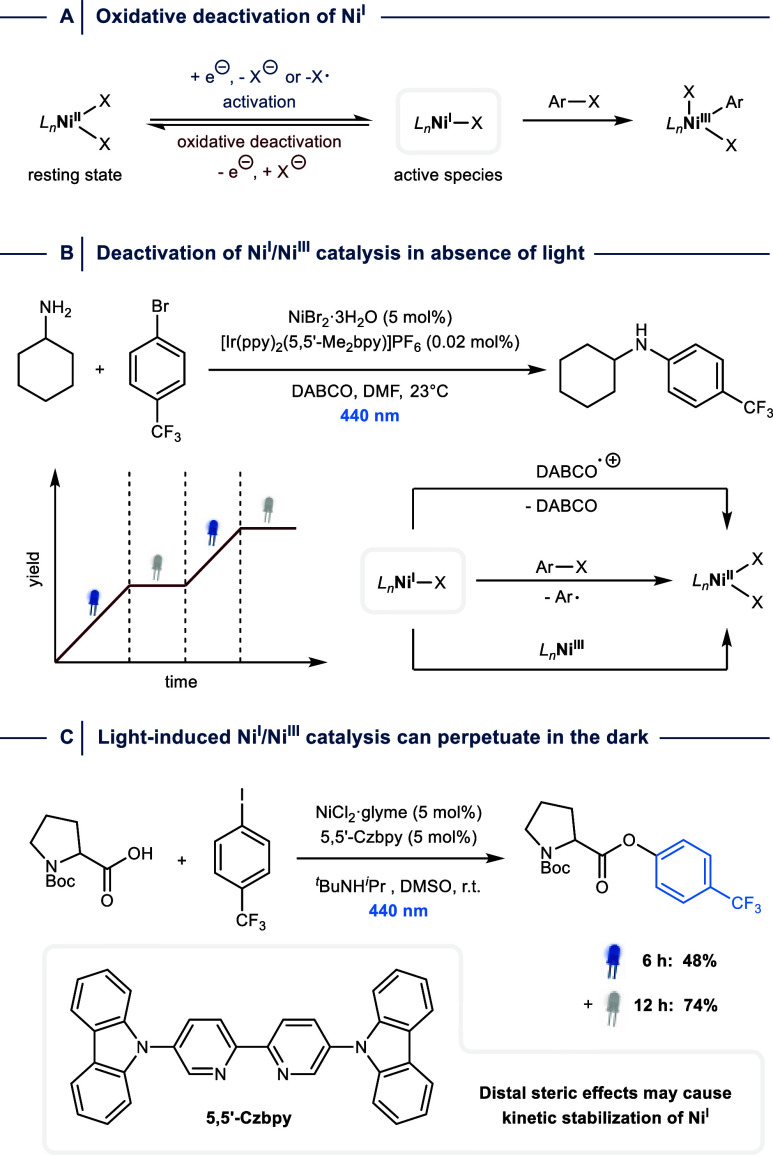
(A) Oxidative deactivation of Ni^I^ generates Ni^II^ resting states. (B) Resting state formation deactivates light-induced Ni^I^/Ni^III^ catalytic cycles. (C) The C­(sp^2^)–O arylation between 4-iodobenzotrifluoride and *N-*Boc-proline perpetuates in the dark when a bpy ligand with bulky substituents in the 5,5′-position is used. r.t. = room temperature. ppy = 2-phenylpyridine. bpy = 2,2′-bipyridine. glyme = 1,2-dimethoxyethane.

A mechanistic study of dual nickel/photoredox catalytic C­(sp^2^)–N bond couplings between amines and aryl bromides in the presence of DABCO showed that although the reduction of Ni^II^ by the photocatalyst is rate-determining, the quantum yield for the Ni catalytic cycle passes the theoretical limit for a one-photon-per-turnover mechanism.^23^ However, coupling reactions in an NMR spectrometer with in situ irradiation showed that the Ni^I^/Ni^III^ catalytic cycle is deactivated within less than 30 s when the light source is switched off (Figure 7B). This is in agreement with steady-state UV/vis absorption measurements, which provided evidence that the buildup of Ni^I^ during catalysis is low. Based on these results, the authors proposed that electron recombination between Ni^I^ and DABCO^•+^, halogen atom abstraction events, and Ni^I^/Ni^III^ comproportionation is responsible for undesired Ni^II^ formation that reduces the overall catalytic efficacy.

More recently, Pieber and colleagues showed that a Ni^I^/Ni^III^ catalytic C­(sp^2^)–O arylation between 4-iodobenzotrifluoride and *N*-Boc-proline can perpetuate in the dark when a photoactive bipyridine ligand substituted with carbazole groups in the 5,5′-position is used (Figure 7C).^53^ While the exact reason for this rare observation remains unclear, kinetic stabilization of Ni^I^ via distal steric protection of the metal center through the ligand field is a plausible explanation.^63^

The slow formation of halide-bridged dimers from monomeric Ni^I^ halide complexes bearing bipyridine ligands constitutes the second major deactivation pathway (Figure 8).^25,28,44,64^ Studies by the groups of Hazari,^64^ Bird, and MacMillan^25^ showed that [(dtbbpy)­NiCl]_2_ and [(dtbbpy)­NiBr]_2_ species are unreactive toward oxidative addition with aryl iodides (Figure 8A). In contrast, monomeric Ni^I^ species readily react with aryl iodides,^25^ which results in a transient Ni^III^ species that undergoes facile comproportionation with Ni^I^.^24^

**Figure 8:**
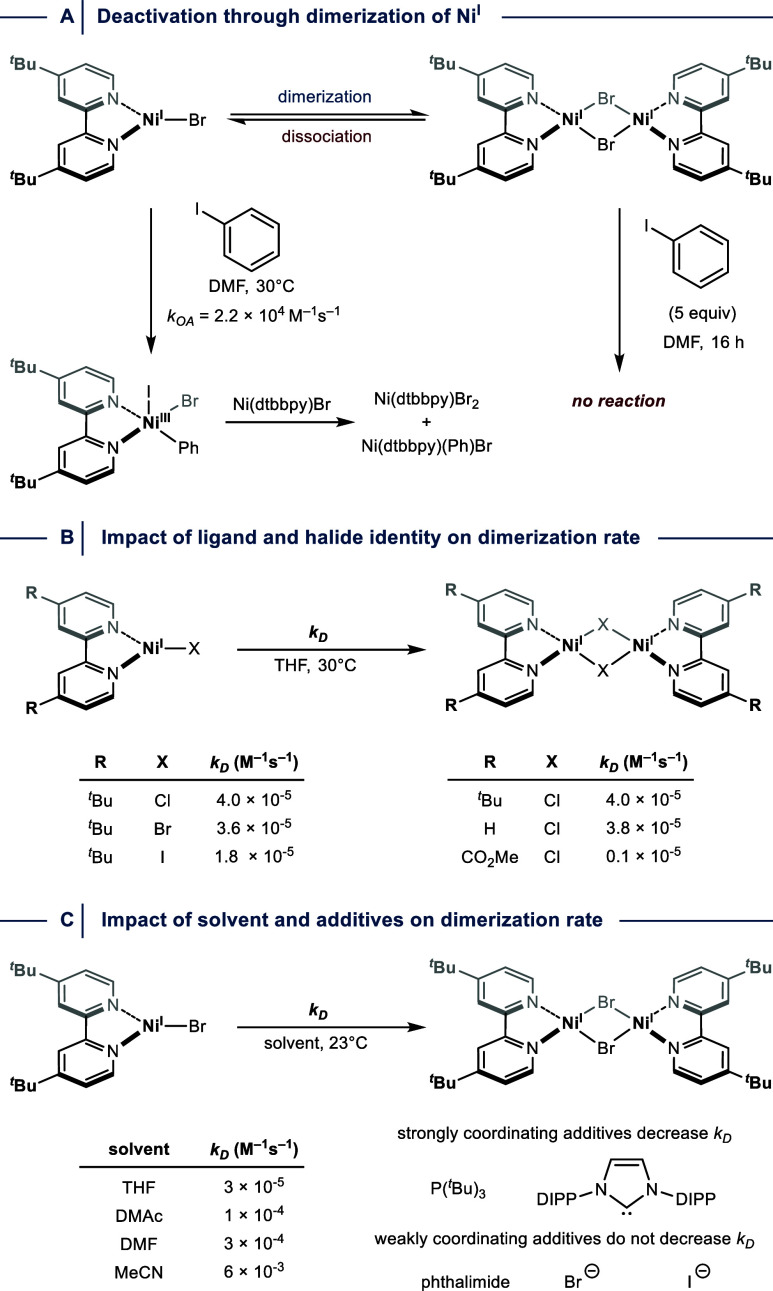
(A) Dimerization of Ni^I^ results in a catalytically inactive species. (B) The halide identity has a significantly smaller effect on dimerization rates than the electronic properties of bipyridine ligands. (C) Solvent and additives impact the dimerization rate. dtbbpy = 4,4′-di-*tert*-butyl-2,2′-dipyridyl. DIPP = 2,6-diisopropylphenyl.

By studying the stability of Ni­(dtbbpy)Cl in THF, Hadt and colleagues found that slow dimerization results in precipitation of [(dtbbpy)­NiCl]_2_.^28^ The authors suggested that the low solubility of dimers in THF suppresses dissociation, which indicates that the solvent choice could be crucial to reduce the thermodynamic driving force for this catalyst deactivation pathway. The same group further demonstrated that the ligands have an impact on the dimerization rate (Figure 8B).^28^ While the halide identity has only a small effect, the electronic properties of bipyridines drastically change the dimerization kinetics. Ni­(dtbbpy)Cl readily dimerizes at room temperature, but low reaction rates were observed when a bipyridine ligand with an electron-withdrawing group in the 4,4′-position was studied as a ligand. Importantly, this careful mechanistic study indicated that the same ligand modifications that are beneficial for the rate of desired OA events between aryl halides and monomeric Ni^I^ species also facilitate dimer formation.

More recently, the Doyle group showed that the dimerization rate of Ni­(dtbbpy)Br indeed spans multiple orders of magnitude depending on the solvent (Figure 8C).^65^ The authors further showed that the addition of strong σ-donor ligands (L), such as phosphines and *N-*heterocyclic carbenes decreases the dimerization rate by rapidly forming 4-coordinate Ni­(dtbbpy)­(L)Br complexes, whereas the addition of weakly coordinating additives (phthalimide or halides) has a negligible impact.

The third reported deactivation pathway in Ni^I^/Ni^III^-catalyzed C­(sp^2^)–heteroatom couplings is the formation of Ni^0^, for example, through over-reduction of Ni^II^ precatalysts (Figure 9A). In the case of C­(sp^2^)–heteroatom cross-coupling reactions that are carried out using *N,N*-bidentate ligands, these low valent nickel species can be stabilized to undergo follow-up processes, such as comproportionation^66,67^ or OA followed by photochemical bond homolysis,^19,44–47^ that regenerate the (pre)­catalyst.

**Figure 9:**
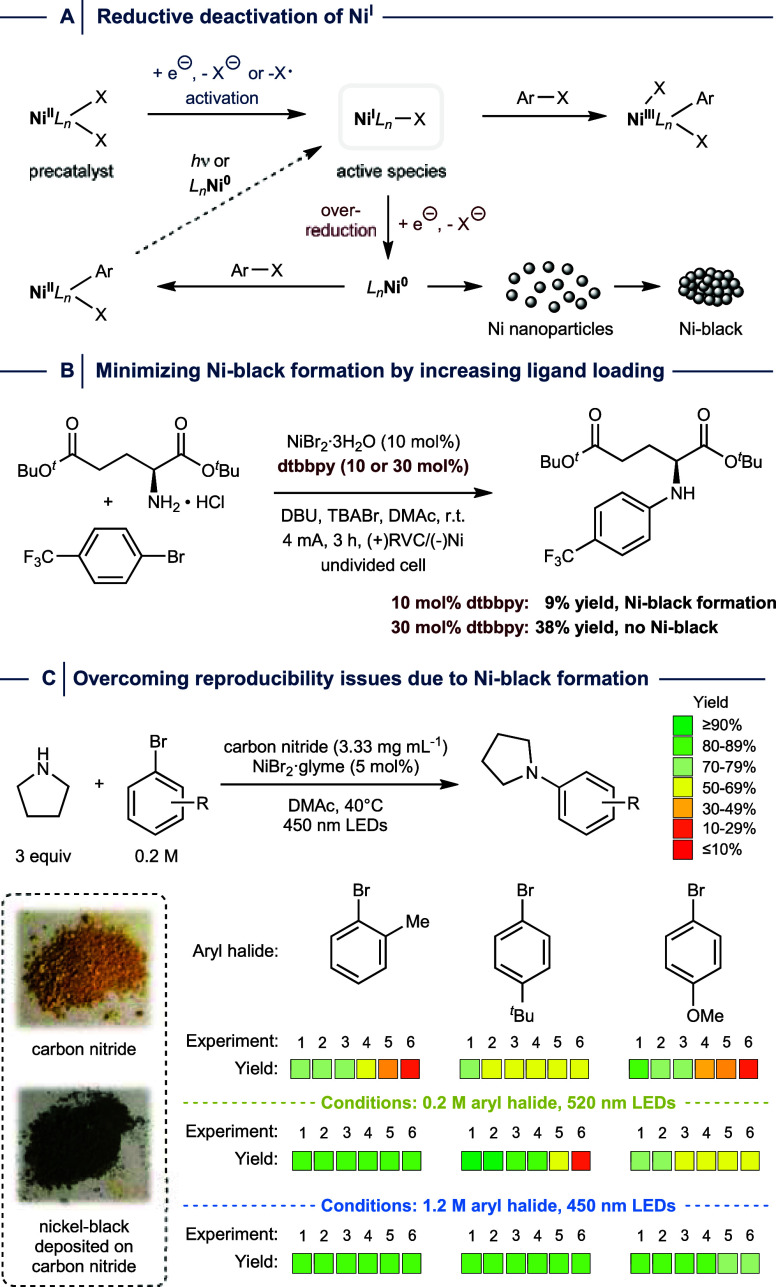
(A) Reductive deactivation of Ni^I^. (B) Nickel-black formation can be avoided by increasing ligand loading in electrochemically driven nickel catalysis. (C) Nickel-black formation causes reproducibility issues in light-mediated C­(sp^2^)–N cross-couplings and can be minimized using longer wavelengths or higher concentrations. dtbbpy = 4,4′-di-*tert*-butyl-2,2′-dipyridyl. r.t. = room temperature. RVC = reticulated vitreous carbon. glyme = 1,2-dimethoxyethane. DBU = 1,8-diazabicyclo[5.4.0]­undec-7-en.

As postulated by Baran and co-workers, accumulation of unligated Ni^0^ species leads to aggregation, resulting in the irreversible formation of nickel-black.^26^ During electrochemically driven nickel-catalyzed C­(sp^2^)–N cross-coupling of an amino acid using 10 mol % of NiBr_2_·3H_2_O and dtbbpy, the authors indeed observed deposition of nickel-black on the cathode and only 10% yield of the desired product (Figure 9B). By increasing the ligand loading to 30 mol %, nickel-black deposition was almost completely suppressed, and a significantly higher yield was obtained.

The Pieber group showed that nickel-black formation is responsible for reproducibility issues in C­(sp^2^)–N cross-couplings between nonactivated aryl halides and amines using a carbon nitride photocatalyst and NiCl_2_ in the absence of *N,N-*bidentate ligands (Figure 9C).^29^ It was concluded that OA between aryl halides and low-valent Ni^I^ species competes with the formation of nickel-black, which deposits on the surface of the heterogeneous photocatalyst.^68–70^ To address this limitation, the authors used a light source that emits longer wavelengths, thereby reducing the propensity for Ni^II^ over-reduction. Alternatively, the authors showed that running these reactions at high concentrations leads to reproducible couplings with high yields, which was ascribed to a higher probability of oxidative addition events. In addition, it was shown that nickel-black formation can be minimized by reducing the loading of iridium polypyridyl complexes in the case of homogeneous dual photoredox/nickel catalysis.

### 3.2 Oxidative Addition

As discussed above, the deactivation of Ni^I^ is closely related to the low OA rates observed between challenging aryl halides and the low-valent, catalytically active transition metal. Hence, overcoming substrate limitations and realizing Ni^I^/Ni^III^ catalyzed C­(sp^2^)–heteroatom couplings using low catalyst loadings will require mechanistically guided strategies that simultaneously reduce undesired side reactions of Ni^I^ and increase its reactivity with electrophiles.

Several reports between 1996 and 2021 established that OA between aryl halides and monomeric Ni^I^ halides can be studied by generating the labile, catalytically active low valent Ni species *in situ* from NiX_2_ precursors in the presence of *N,N-*bidentate ligands through chemical,^71^ electrochemical,^19,26,72–75^ photocatalytic,^76^ or radiolytic reduction (Figure 10A).^25^ These approaches allow for kinetic studies to investigate the impact of ligand and electrophile variations on the mechanism by which the aryl halide’s C–X bond is activated. More specifically, Hammett analysis can provide evidence whether OA occurs through a process that resembles nucleophilic aromatic substitution (S_N_Ar-type: ρ ∼ 5;^77^), a concerted addition of the aryl halide involving a three-membered transition state (Con-type: ρ ∼ 2;^78^), single-electron transfer activation (SET-type: ρ ∼ 4;^79^), or a halogen atom abstraction mechanism (HAA: ρ ∼ 1 ^80^) (Figure 10B).^81^

**Figure 10:**
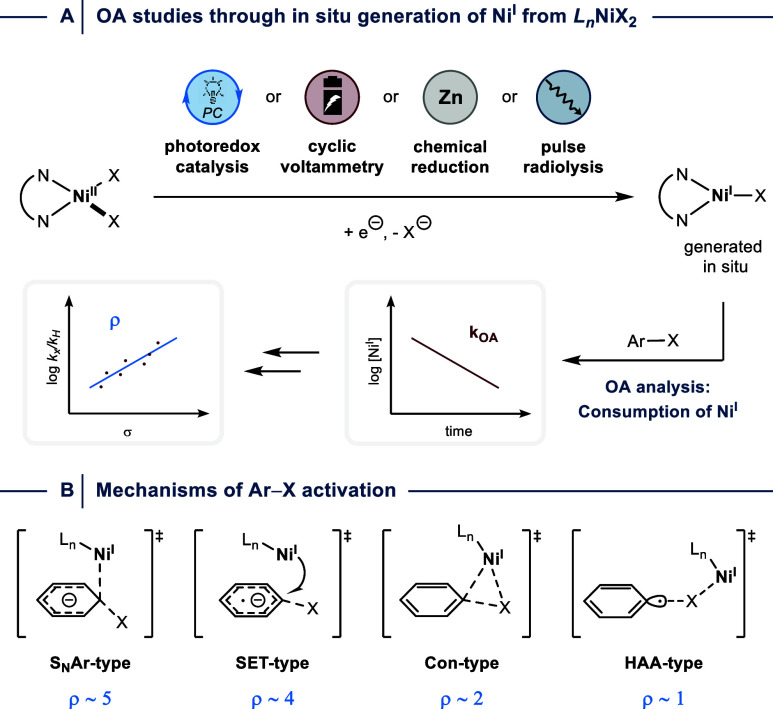
(A) Kinetic analysis of oxidative addition (OA) between Ni^I^ and aryl halides. (B) Possible mechanisms of Ar–X activation during OA. SET = single electron transfer. Con = concerted. HAA = halogen atom abstraction.

For example, the groups of MacMillan and Bird employed pulse radiolysis to generate a Ni^I^ species from Ni­(dtbbpy)­Br_2_ in the presence of aryl iodides using DMF, which generates solvated electrons upon ionization, and 1,3-dicyanobenzene as an electron-transfer mediator that ensures efficient Ni^I^ generation and minimizes a SET event with the aryl iodide (Figure 11A).^25^ Analysis of OA processes using aryl iodides with electronically different substituents in the *para*-position was achieved by monitoring the decay of the 650 nm absorption feature of Ni­(dtbbpy)Br using time-resolved optical absorption spectroscopy and provided OA rate constants (Figure 11B). A ρ-value of +1.3 was obtained by plotting log­(*k*_X_ */k*_H_),^82^ which is indicative of Con- or HAA-type OA. Moreover, the authors performed OA studies at different temperatures using ethyl 4-iodobenzoate. Eyring analysis of the resulting data allowed determining enthalpic (Δ*H* ^⧧^ = 5.8 ± 0.2 kcal·mol^–1^) and entropic (Δ*S* ^⧧^ = −15.5 ± 0.3 cal·mol^–1^·K^–1^) contributions to the overall reaction barrier (Δ*G*_298K_^⧧^ = 10.4 ± 0.4 kcal·mol^–1^). In addition, these experiments suggested that sample degradation or a change in the OA mechanism occurs above ∼50 °C.

**Figure 11:**
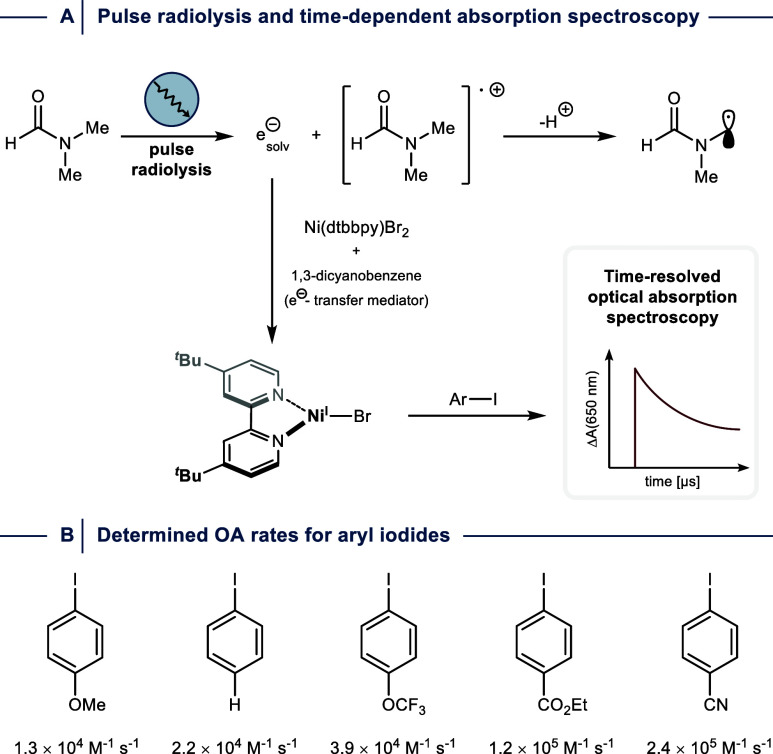
(A) Principle of OA rate determination between Ni^I^ and aryl iodides using pulse radiolysis and time-resolved optical absorption spectroscopy. (B) Determined OA rates for electronically different aryl iodides. dtbbpy = 4,4′-di-*tert*-butyl-2,2′-dipyridyl.

Cyclic voltammetry (CV) experiments can correlate the electrochemical generation of Ni^I^ (E-step) with their chemical consumption (C-step) in the presence of aryl halides (EC mechanism).^75,83^ An important aspect for such CV measurements is that the choice of solvent (DMAc) and supporting electrolyte (tetrabutylammonium bromide) is crucial for obtaining high-quality data.^19,75^ This can be explained by studies from Diao and co-workers, which showed that the presence of coordinating species, such as bromide anions, stabilizes Ni^I^ complexes bearing redox-active ligands by forming four-coordinate square-planar low-spin Ni^II^ species coordinated to radical anion ligands (see Figure 1B).^49^

Pieber and co-workers recently used the CV approach to provide qualitative evidence that OA between a Ni^I^ halide complex bearing a carbazole-substituted bipyridine and an electron-poor aryl bromide is feasible, while an electron-rich aryl bromide does not react efficiently (Figure 12A).^19^ In comparison to the CV of the Ni^II^ precatalyst, a decrease in reversibility was observed in the presence of the activated electrophile as indicated by the lowering of the return peak’s intensity (B), and the emergence of a new species (C), that was assigned to a Ni^II^(aryl) species formed by facile reduction of the Ni^III^ OA complex.^75^

**Figure 12:**
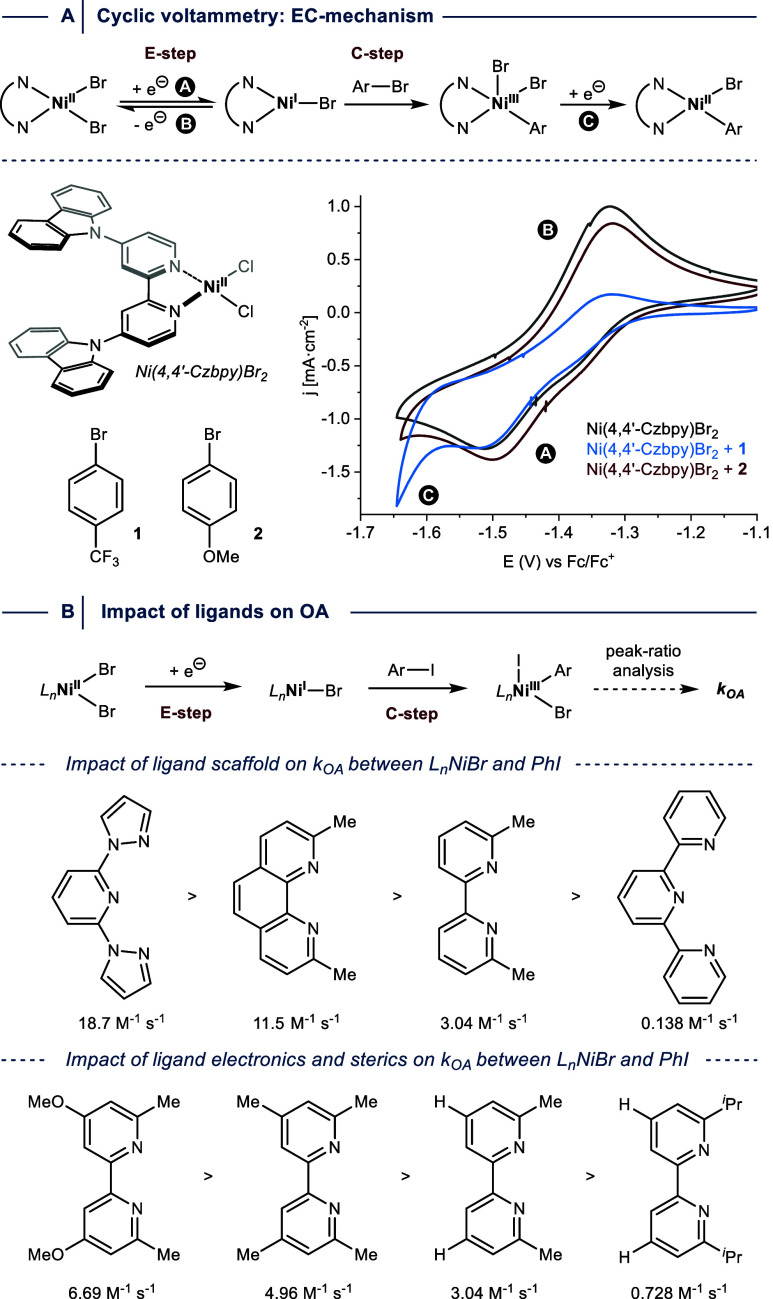
(A) Oxidative addition (OA) studies between Ni^I^ and aryl iodides using cyclic voltammetry. (B) Impact of ligands on OA reactivity.

The above-discussed example shows that the CV approach is operationally more straightforward for OA studies compared to the combination of pulse radiolysis and time-resolved optical absorption spectroscopy. However, speciation processes of Ni^II^ and disproportionation events involving Ni^I^ often lead to overlapping peak responses, which prevent qualitative and quantitative analysis in the case of bipyridine ligands that are most common in Ni^I^/Ni^III^-catalyzed C­(sp^2^)–heteroatom cross-couplings, such as dtbbpy.^26,71,75^ The groups of Doyle and Sigman circumvented this problem by studying bipyridine (bpy) and phenanthroline (phen) ligands bearing methyl groups adjacent to the ligand’s coordinating *N-*atoms that shield the metal center.^75^ In the case of tridentate ligands (terpyridines (terpy) and 2,6-bis­((1H-pyrazol-1-yl)­methyl)-pyridines (bpp)), this stabilization was not required, which was attributed to the increased coordinative saturation.

Together, this allowed the authors to determine rate constants for OA processes involving 44 different aryl halides and 16 different ligands through peak-ratio analysis (Figure 12B). A comparison of the impact of ligand scaffolds using phenyl iodide showed that the fastest OA rate is achieved using a bpp derivative, whereas a terpyridine ligand results in the slowest reaction. Hammett-type analysis using electronically different aryl iodides resulted in ρ-values between +1 and +2 for all ligand scaffolds. A comparison of 6,6′-dimethyl-2,2′-bipyridine ligands that additionally have electronically different substituents in the 4,4′-position demonstrated that electron-rich ligands are beneficial for OA. This trend is similar across all ligand classes. Further, increasing the steric bulk adjacent to the ligand’s coordinating *N-*atoms inhibits OA. Similarly, *ortho*-substituted aryl iodides react more slowly than *para*-substituted analogs, which underscores the steric sensitivity of the OA step. Overall, the authors conducted >200 rate measurements. Together with DFT computed parameters, this experimental data was subjected to multivariate linear regression analysis, which led to the conclusion that the use of bpy, phen, and terpy ligands results in a Con-type OA process, while a halogen atom abstraction mechanism is operative when bpp ligands are used.

In an alternative approach, Hadt and colleagues demonstrated that Ni^I^ complexes that do not bear substituents for steric protection of the metal center can be almost quantitatively generated from Ni­(^R^bpy) aryl halide complexes by air- and moisture-free irradiation at 370 nm (Figure 13A).^28^ The resulting species are sufficiently stable in solution, which enables characterization using optical and electron paramagnetic spectroscopy, and allows assessing their OA reactivity by analyzing the depletion of their MLCT absorption bands in the presence of aryl halides over time. The authors demonstrated that facile reactions occur in the presence of 2-bromo-toluene, but also unveiled that aryl chlorides, which are typically unreactive in Ni^I^/Ni^III^-catalyzed C­(sp^2^)–heteroatom cross-couplings, engage in OA processes. While the original interpretation of the data obtained during Hammett-type analysis suggested an S_N_Ar-type mechanism (ρ ∼ 5) when ln­(*k*_X_/*k*_H_) was plotted vs sigma, Doyle and co-workers recently pointed out that plotting log­(*k*_X_/*k*_H_) would suggest that a Con- or HAA-type mechanism (ρ ∼ 2) is operative.^65^ Regardless of the underlying mechanism, the authors showed that the halide identity of the Ni­(^R^bpy)­X catalyst has only a little effect on OA rates, and that electron-donating groups at the 4,4′-position of bpy are crucial for high reactivity. Theoretical investigations correlated this experimental observation with the substituent impact on the energy of the metal’s *3d­(z^2^)* orbital (Figure 13B).

**Figure 13:**
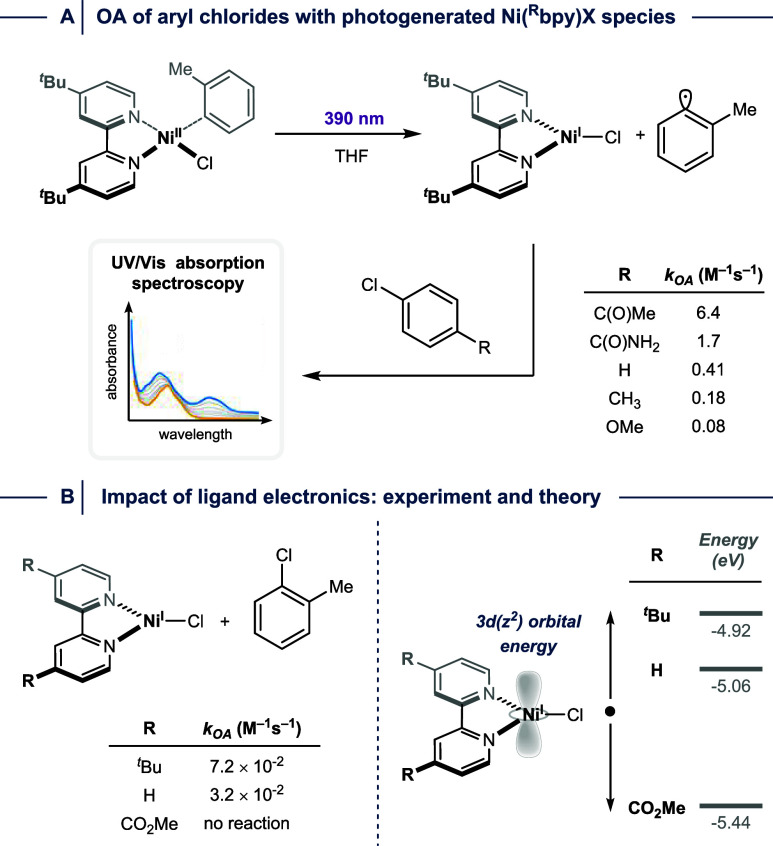
(A) Photogeneration of Ni^I^ from Ni­(^R^bpy) aryl halide complexes enables studying oxidative addition (OA) kinetics with aryl chlorides. (B) The impact of ligand modifications on OA rates can be correlated with the metal’s *3d­(z* *^2^)* orbital energy.

All studies described above rely on *in situ* generated Ni^I^ intermediates rather than well-defined species that can be fully characterized. Recently, the Doyle group presented a straightforward approach to prepare isolable Ni^I^ bipyridyl complexes that are stabilized through coordination of *E*-stilbene (Figure 14A).^65^ The olefin dissociates upon dissolving the four-coordinate species, which provided access to reactivity studies of N­(^R^bpy)­X under various conditions. Using this approach, the authors studied OA kinetics with aryl chlorides in THF using UV/Vis absorption spectroscopy, which resulted in comparable results to those determined through the photogenerated species discussed above (see Figure 13).

**Figure 14:**
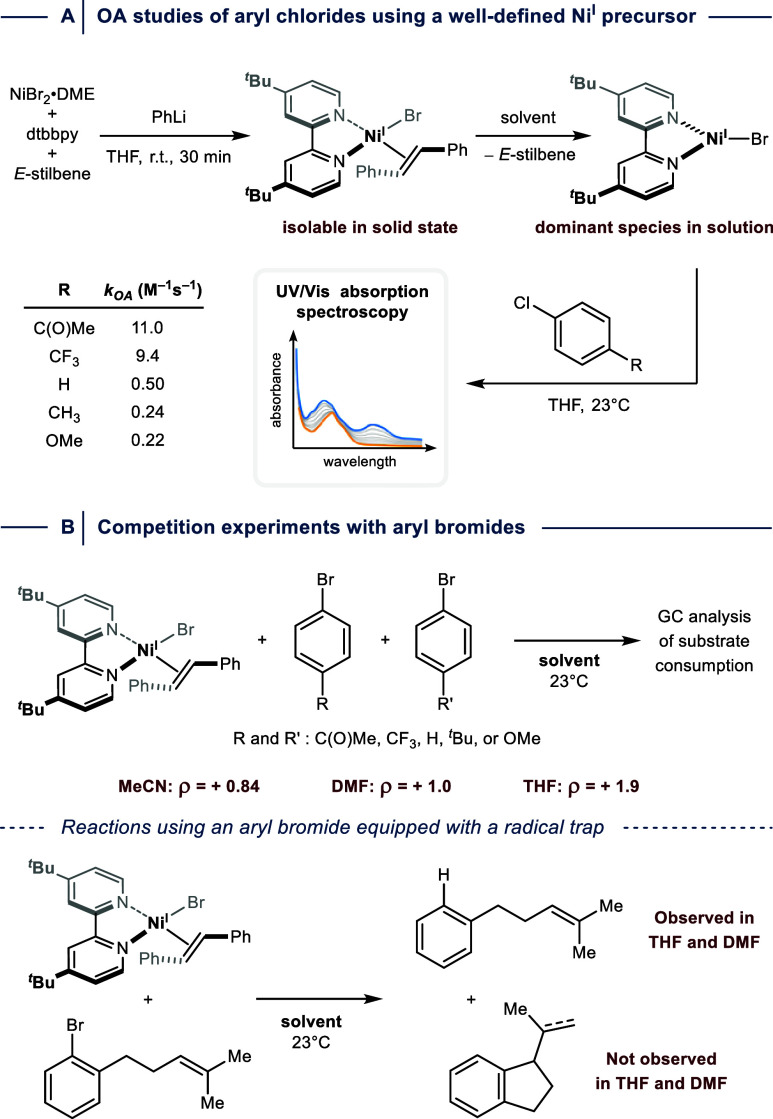
Well-defined, isolable Ni^I^ bipyridyl complex streamlines mechanistic studies. (A) Oxidative addition (OA) rate determination between Ni^I^ and aryl chlorides using UV/Vis absorption spectroscopy. (B) Competition experiments enable Hammett-type analysis for aryl bromides. DME = 1,2-dimethoxyethane. dtbbpy = 4,4′-di-*tert*-butyl-2,2′-bypiridine r.t. = room temperature.

Similar to the work by the Hadt group, OA with aryl bromides was reported to be too fast for obtaining rate constants. However, analyzing substrate consumption during competition experiments using different solvents enabled determining relative rates for Hammett-type studies (Figure 14B). The determined ρ-values of +1.0 (DMF) and +0.84 (MeCN) are indicative of an HAA-type mechanism, whereas a Con-type mechanism seemed plausible in THF (ρ = +1.9). However, it is difficult to clearly distinguish between the radical and nonradical pathways due to similar ρ-values. Hence, the authors conducted experiments with an aryl bromide bearing a radical trap. Using THF and DMF as solvents, no cyclization product was detected, which is most consistent with a Con-type mechanism. Notably, the authors mentioned that a rapid in-cage radical recombination with Ni cannot be fully excluded at this stage.

## 4 Emerging Strategies to Address Limitations in Ni^I^/Ni^III^ Catalysis

### 4.1 Reaction Temperature

The mechanistic OA studies discussed above provide striking evidence that stereoelectronic factors of ligands and electrophiles impact OA between Ni^I^ halide species and aryl halides. Electron-rich ligands increase the nucleophilicity of Ni^I^, but OA rates of aryl chlorides and electron-rich aryl bromides are still not sufficiently high to enable broadly applicable C­(sp^2^)–heteroatom cross-couplings using these substrates.

DFT calculations suggested that OA is rate-determining in Ni^I^/Ni^III^ catalysis using challenging electrophiles with calculated Gibbs free-energy barriers of ≈23 kcal mol^–1^ for OA between Ni­(^R^bpy)­X and 2-chlorotoluene at room temperature.^28^ It is therefore not surprising that empirical data from several methodology studies suggest that the electrophile scope can be expanded when reactions are carried out at elevated temperatures. For example, during the development of a continuous flow protocol for dual photoredox/nickel catalyzed (hetero)­aryl aminations, Buchwald and co-workers found that the yield of the cross-coupling reaction between a primary amine and bromobenzene can be significantly improved using a reaction temperature of 80 °C (Figure 15A).^84^ The Xue group reported a beneficial thermal effect for light-mediated C­(sp^2^)–N couplings using similar substrates. More specifically, the authors showed that a small distance between the light source and the reaction vial increases not only the photon density, but also raises the reaction temperature to 85 °C, resulting in higher yields.^52^ This reaction temperature proved also most efficient for the synthesis of anilines through the coupling of aryl halides with ammonium salts using the same catalytic approach.^85^

**Figure 15:**
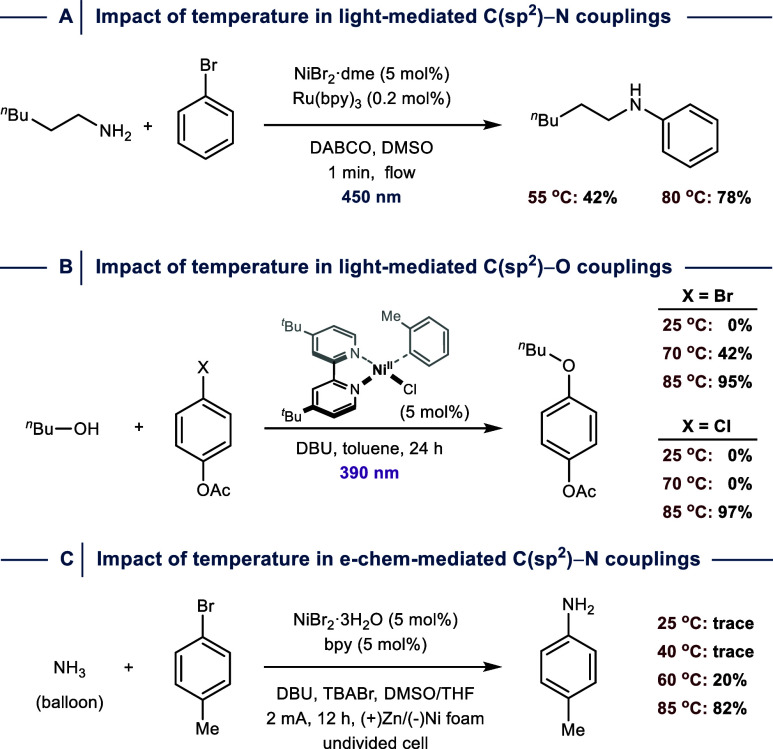
Impact of reaction temperature in light-mediated (A) C­(sp^2^)–N and (B) C­(sp^2^)–O cross-coupling reactions. (C) Impact of reaction temperature in electrochemically enabled C­(sp^2^)–N cross-coupling. dme = 1,2-dimethoxyethane. bpy = 2,2′-bipyridine. DABCO = 1,4-diazabicyclo[2.2.2]­octane. DBU = 1,8-diazabicyclo[5.4.0]­undec-7-en.

More recently, the same group studied the impact of reaction temperature on light-mediated C­(sp^2^)–O couplings between a primary alcohol and both an electron-poor aryl bromide and chloride (Figure 15B).^49^ The authors showed that no coupling products are obtained when the reactions are carried out at 25 °C. In the case of the aryl bromide, product formation was observed at 70 °C, whereas 85 °C were necessary for initiating cross-couplings using the aryl chloride.

These literature examples clearly show that the reaction temperature is a crucial parameter in light-induced Ni^I^/Ni^III^ catalyzed C­(sp^2^)–heteroatom cross-couplings that needs to be studied in greater detail. However, these reports also underscore the importance of the experimental setup. Light-mediated reactions are usually carried out with home-built setups using LEDs from a variety of vendors that have different specifications. Emission spectra, photon output, the distance between the light source and the reaction vial, as well as all other technical aspects (fan cooling, etc.) will impact the outcome of Ni^I^/Ni^III^-catalyzed cross-couplings, and many other photochemical transformations.^86^ The standardization of photochemical reactors using dedicated, commercial equipment might be an ideal solution, but is unlikely to happen due to the low prices of self-made setups. Hence, accurate descriptions of light sources, reactor arrangements, and the exact conditions are not only crucial for reproducing reported data, but also key for interpreting outcomes of reactions properly.^87,88^

A beneficial thermal effect has also been demonstrated for the electrochemically mediated nickel-catalyzed synthesis of anilines through the direct coupling of aryl halides with gaseous ammonia (Figure 15C).^89^ Studies of the reaction using 4-bromotoluene as electrophile resulted in trace amounts of the product at temperatures below 40 °C. A coupling attempt at 60 °C resulted in 20% of the desired product. Further raising the reaction temperature to 85 °C increased the yield of the desired aniline to 82%. The authors assumed that elevated temperatures are necessary to avoid formation of stable, catalytically inactive nickel–ammonia complexes, or to liberate NH_3_ from such an intermediate.

### 4.2 Precise Control of Redox Conditions

A benefit of electrochemical methods compared to other approaches that enable Ni^I^/Ni^III^-catalyzed C­(sp^2^)–heteroatom cross-couplings is the ability to precisely tune the redox potentials to catalysis needs. Baran and co-workers leveraged this advantage to develop a protocol for the O-arylation of alcohols that tolerates oxidatively labile groups, such as tertiary amines (Figure 16A).^89^ The authors showed that previously developed conditions for C­(sp^2^)–O couplings via the combination of nickel and photoredox catalysis do not provide the desired products using these substrates.^90^ It was suggested that this results from the precise control of redox conditions using the electrochemical approach, which eventually favors Ni^III^ formation over competitive tertiary amine oxidation.

**Figure 16:**
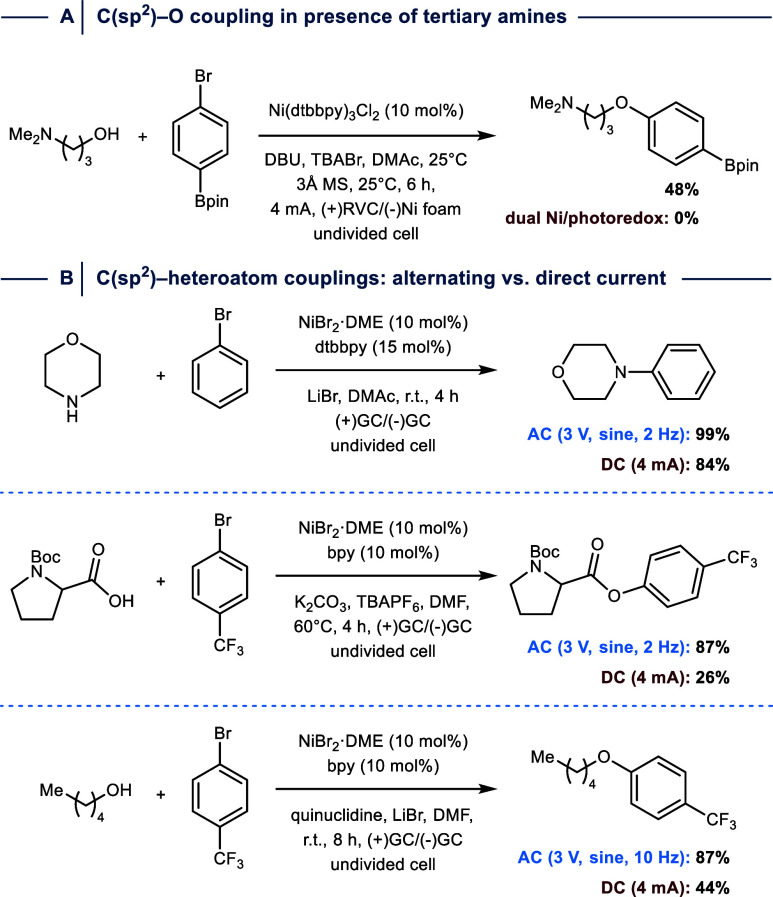
(A) Electrochemistry enables Ni-catalyzed C­(sp^2^)–O couplings of alcohols that contain tertiary amine groups. (B) Electrochemically mediated Ni-catalyzed C­(sp^2^)–heteroatom couplings using alternating current (AC) give better yields compared to direct current (DC) experiments. Dtbbpy = 4,4′-di-*tert*-butyl-2,2′-bypiridine. MS = molecular sieves. RVC = reticulated vitreous carbon. r.t. = room temperature. GC = glassy carbon. DBU = 1,8-diazabicyclo[5.4.0]­undec-7-en.

Electrochemical synthesis is typically carried out using direct current (DC) electrolysis, however, several recent examples indicate that alternating current (AC) electrolysis, which applies rapidly switching electrode polarity, can enable improved reactivity by repeatedly inverting oxidation and reduction environments at the electrode surface.^91,92^ Semenov and co-workers compared these two approaches for several Ni^I^/Ni^III^-catalyzed C­(sp^2^)–heteroatom cross-couplings under otherwise identical conditions, and found that yields using the AC approach are generally higher (Figure 16B).^93^ Encouraged by these results, the groups of Carvallho and Jones developed a flow method that provides facile access to small molecule libraries using AC conditions, which were crucial for preventing electrode passivation, obtaining high selectivities, and avoiding the use of supporting electrolytes.^94^ More recently, Luo and colleagues performed a comprehensive inverstigation of AC-mediated Ni^I^/Ni^III^-catalyzed C­(sp^2^)–N bond formations to study the impact of AC frequency on cross-coupling selectivity.^95^ The authors demonstrated that optimizing this parameter for each substrate is crucial to avoid formation of off-cycle species that lead to undesired C­(sp^2^)–(Cp^2^) homocouplings.

### 4.3 Alternative Electrophiles

As discussed throughout this article, challenging electrophiles such as electron-rich aryl bromides and aryl chlorides often fall outside the scope of C­(sp^2^)–heteroatom cross-coupling methods through Ni^I^/Ni^III^ catalysis. The Cornella and Ritter groups elegantly addressed this limitation by employing aryl thianthrenium salts,^96^ which have recently been established as an alternative class of electrophiles for many bond formations.^97^ Synthesis of these reagents is conveniently achieved through site-selective, aromatic C–H functionalization of electron-rich arenes with thianthrene-5-oxide (Figure 17A).^98^

**Figure 17:**
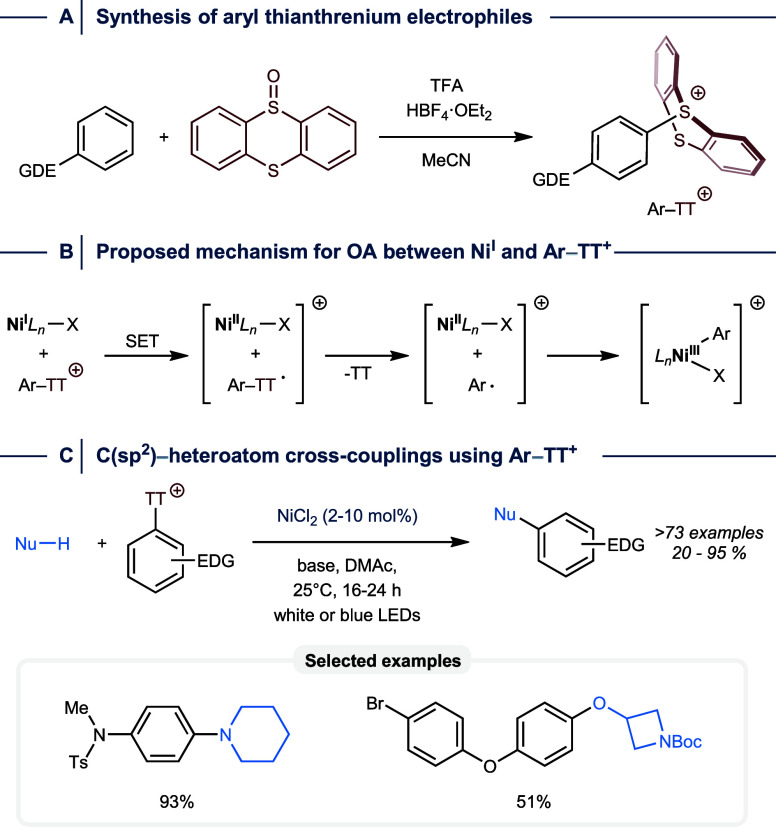
(A) Synthesis of aryl thianthrenium salts. (B) Proposed mechanism of oxidative addition (OA) between Ni^I^ and aryl thianthrenium salts. (C) Cross-coupling conditions and selected examples.

In contrast to aryl halides that are activated by a concerted mechanism or a halogen atom abstraction OA process, aryl thianthrenium salts were proposed to engage in a single-electron reduction in the presence of Ni^I^ (Figure 17B). The resulting thianthrenium radical undergoes facile bond homolysis, leading to an aryl radical that recombines with Ni^II^ to produce the desired Ni^III^ oxidative addition complex. Substituents at these electrophiles play a minor role in the OA rate because the electronic structure of the thiantrenium unit primarily governs the redox properties of these electrophiles. This strategy has been successfully applied to the light-mediated C­(sp^2^)–N, C­(sp^2^)–O, and C­(sp^2^)–S cross-couplings catalyzed by NiCl_2_ without added ligands and resulted in good to excellent yields using several electron-rich electrophiles (Figure 17C). However, generalization of this approach is hampered by the low selectivity of reactions with e^–^-poor derivatives that are also difficult to prepare.^98^

### 4.4 Additives

Numerous conditions that were carefully optimized for specific substrate combinations have been published over the past decade for Ni^I^/Ni^III^-catalyzed C­(sp^2^)–heteroatom cross-couplings. This includes the various approaches to initiate the catalytic cycle (photocatalysis, electrochemistry, etc.) discussed in this perspective, different Ni^II^ precatalysts, ligands, and solvents. In contrast to canonical Pd^0^/Pd^II^-catalysis, one of the benefits of Ni^I^/Ni^III^-catalysis is that insoluble inorganic bases, such as potassium *tert*-butanolate, are not necessary. Instead, organic bases, such as DABCO, DBU, or quinuclidine, are often employed. These additives were proposed to have multiple roles beyond acting only as a Brønsted base, including serving as ligands,^29^ sacrificial electron donors,^24^ or electron shuttles.^76^

A systematic study classified nucleophiles depending on the requirement of specific additives in Ni^I^/Ni^III^-catalyzed C­(sp^2^)–heteroatom cross-couplings through the merger of photoredox and ligand-free nickel catalysis.^22^ Nucleophiles of the first group, such as thiols and amines, readily coordinate to Ni^II^ salts and engage in additive-free cross-couplings with electron-poor aryl bromides. Group two nucleophiles (e.g., sulfoximines) also coordinate to Ni^II^, but catalysis benefits from the addition of DABCO, which assists in forming a catalytically competent species and neutralizes HBr that is generated during cross-coupling catalysis. Group three nucleophiles, such as sulfonamides, do not coordinate to the Ni^II^ precatalyst and require the addition of sterically demanding amines that act as ligands but do not engage in cross-couplings under these conditions.^99^ Aliphatic alcohols belong to group four nucleophiles, which require the addition of a sufficiently strong base, such as 1,1,3,3,-tetramethylguanidine (TMG), to induce interaction between the nucleophile and the Ni salt upon deprotonation.

More recently, the same group found that the addition of Brønsted acids facilitates C­(sp^2^)–S cross-coupling between aliphatic thiols and aryl bromides (Figure 18A).^100^ While additive-free conditions suffered from a significant induction period, facile catalysis is immediately achieved in the presence of substoichiometric amounts of HBr. Mechanistic studies provided evidence that the acidic conditions prevent the formation of nickel polythiolate complexes that are likely catalytically inactive and detrimental for photochemical processes due to their dark color, which leads to an inner filter effect. Increasing the amount of HBr further harnessed bromoaniline derivatives as electrophiles by converting the electron-rich substituent into an electron-withdrawing moiety through protonation (Figure 18B).^101^

**Figure 18:**
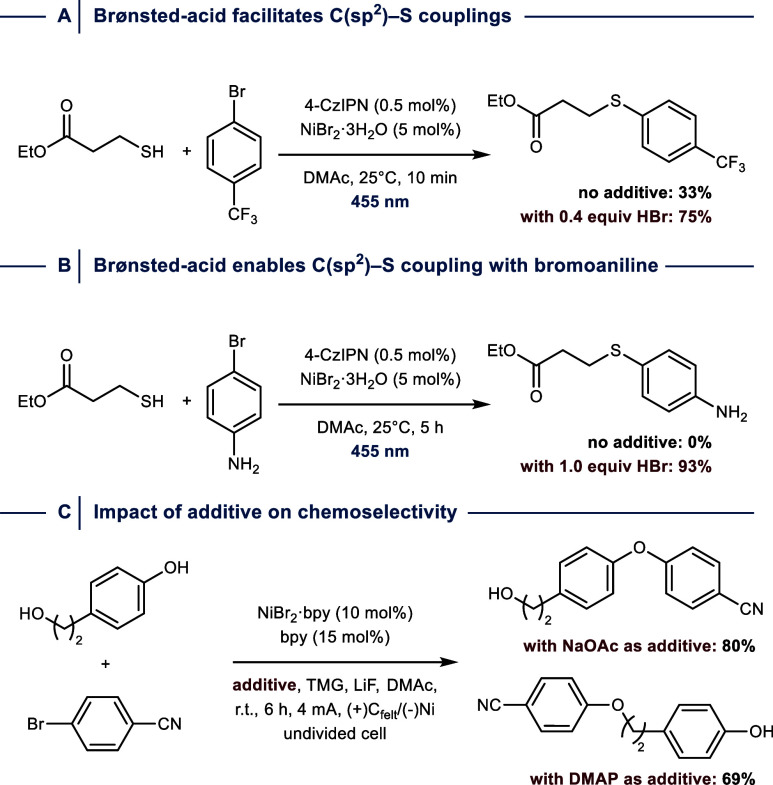
(A) Brønsted acid additive facilitates C­(sp^2^)–S cross-coupling by preventing Ni polythiolate formation and (B) enables reactions with bromoanilines through protonation. DMAc, dimethylacetamide. HBr, hydrobromic acid. (C) The choice of additive impacts the chemoselectivity of C­(sp^2^)–S cross-couplings. 4-CzIPN = 1,2,3,5-tetrakis­(carbazol-9-yl)-4,6-dicyanobenzene. bpy = 2,2′-bipyridine. TMG = 1,1,3,3-tetramethylguanidine. DMAP = 4-dimethylaminopyridine.

Notably, the choice of additives was shown to have a profound impact on the chemoselectivity of electrochemically mediated Ni^I^/Ni^III^ catalyzed C­(sp^2^)–O cross-couplings between aryl bromides and nucleophiles that contain a phenolic and aliphatic alcohol functionality (Figure 18C).^102^ While the addition of substoichiometric amounts of NaOAc selectively promotes phenol arylation, use of DMAP inverts the selectivity toward aryl alkyl ether bond formation. Mechanistic studies indicate that the acetate anion acts a base, accelerates ligand exchange with phenols, and promotes reductive elimination through the formation of a six-membered transition state. Replacing NaOAc with DMAP selectively suppresses this pathway, which results in couplings with the more nucleophilic aliphatic alcohol.

### 4.5 Ligand Design

As discussed in Chapter 3.2, electron-rich bipyridine ligands facilitate oxidative addition between aryl halides and Ni^I^. However, only a handful of bipyridine ligands, such as dtbbpy, are typically used in Ni^I^/Ni^III^-catalyzed C­(sp^2^)–heteroatom couplings. In an effort to overcome substrate limitations through the design of new ligand architectures, Pieber and colleagues recently demonstrated that 4,4′-diphenylamino-2,2′-bipyridine (dpabpy) results in significantly higher catalytic activity than common, commercially available bipyridines and allows using catalyst loadings as low as 100 ppm.^56^ This was ascribed to a combination of an increased nucleophilicity of Ni^I^, caused by the electron-donating amino-groups, and the low redox potential of the ligand, which facilitates stabilization through the formation of a ligand-centered radical (Figure 19). Systematic investigations showed that the combination of Ni­(dpabpy)­Cl_2_ as precatalyst and Barton’s base (BTMG) as additive results in a highly general catalytic system for C­(sp^2^)–heteroatom bond formations. The scope includes couplings of electron-rich aryl bromides with *N-, O-, S-*, and *P-*nucleophiles. A photocatalyst was not necessary because BTMG serves the dual role of enabling precatalyst activation with visible light and harnessing a broad range of nucleophiles, including challenging tertiary alcohols and *α,α,α*-trisubstituted amines that were previously deemed unsuitable substrates for light-mediated Ni^I^/Ni^III^ catalysis.

**Figure 19:**
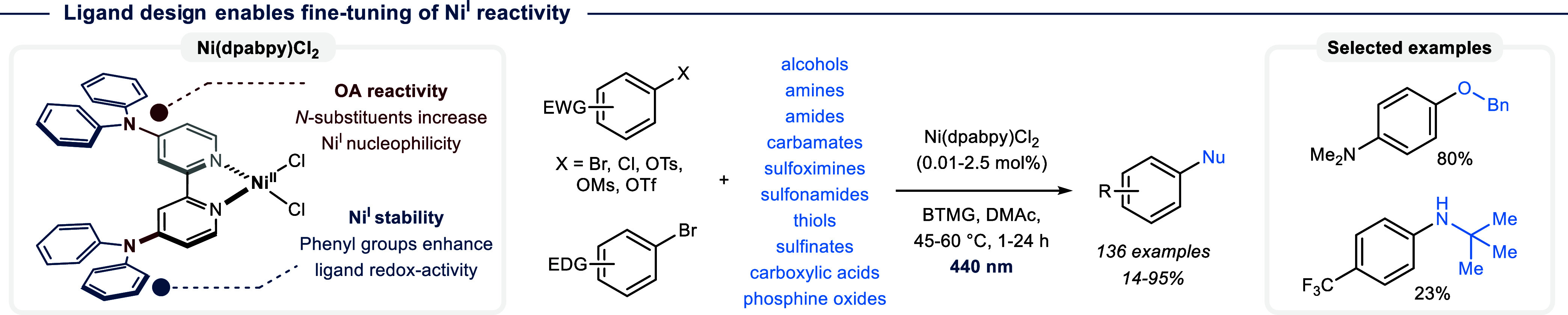
Ligand design enables C­(sp^2^)–heteroatom cross-coupling at low nickel catalyst loadings, using electron-rich aryl bromides, and sterically encumbered nucleophiles. dpabpy = 4,4′-diphenylamino-2,2′-bipyridine. EWG = electron withdrawing group. EDG = electron donating group. BTMG = 2-*tert*-butyl-1,1,3,3-tetramethylguanidin.

## 5 Conclusion and Outlook

Major advances in Ni^I^/Ni^III^-catalyzed C­(sp^2^)–heteroatom cross-couplings include the development of straightforward strategies to access the paramagnetic species from bench-stable Ni^II^ precatalysts, insights into the mechanisms of catalyst deactivation and the rate limiting OA step, and strategies that leverage this knowledge to overcome bottlenecks in the field. Despite the immense progress highlighted in this article, several challenges remain that must be addressed in order to mature Ni^I^/Ni^III^-mediated catalysis into a broadly applicable approach that can serve as a real alternative for canonical Pd^0^/Pd^II^ catalysis. This includes limitations of the electrophile scope. Although electron-rich and *ortho*-substituted aryl chlorides do react with stoichiometric amounts of Ni^I^, the OA rates are not sufficiently high to allow for efficient catalytic methods using these substrates. Sterically encumbered nucleophiles were only shown to undergo couplings using activated electrophiles, and there is no detailed understanding for this observation. From the above-discussed examples, it becomes clear that the choice of additives, reaction temperature and redox conditions has a major impact on the success and selectivity of cross-coupling reactions, but the exact reasons for these observations are yet poorly understood.

Maturing Ni^I^/Ni^III^-mediated catalysis into a broadly applicable approach that can serve as a real alternative for canonical Pd^0^/Pd^II^ catalysis will undoubtedly require a protocol that enables harnessing sterically demanding nucleophiles and electrophiles with low reactivity, such as aryl chlorides and pseudohalides. From a medicinal chemistry standpoint, it will be important to study whether the Ni^I^/Ni^III^ manifold can provide a straightforward approach to harness drug-like starting materials and challenging heteroaryl halides that require extensive screenings of ligands and conditions in the case of palladium catalysis.^103–105^ To achieve this, comprehensive mechanistic studies of factors that impact reactivity of low valent Ni^I^ species and a better understanding of the entire Ni^I^/Ni^III^ manifold, including undesired off-cycle events, are required. This knowledge will be crucial to inform the development of next-generation ligands, the selection of additives, and reaction conditions. It is expected that the implementation of enabling technologies, such as machine learning and high-throughput experimentation, will additionally streamline and guide the evolution of Ni^I^/Ni^III^ catalysis methods, eventually resulting in a universally applicable protocol for C­(sp^2^)-heteroatom couplings.
